# Highly Efficient JR Optimization Technique for Solving Prediction Problem of Soil Organic Carbon on Large Scale

**DOI:** 10.3390/s24227317

**Published:** 2024-11-15

**Authors:** Harsh Vazirani, Xiaofeng Wu, Anurag Srivastava, Debajyoti Dhar, Divyansh Pathak

**Affiliations:** 1 School of Aerospace, Mechanical and Mechatronic Engineering, University of Sydney, Sydney, NSW 2050, Australia; hvaz8919@uni.sydney.edu.au; 2 Atal Bihari Vajpayee Indian Institute of Information Technology and Management Gwalior, Gwalior 474015, India; profanurag@gmail.com (A.S.); debajyotidhar111@gmail.com (D.D.); divyanshpathak1964@gmail.com (D.P.)

**Keywords:** GeoBlendMDWC, optimization, regression, soil organic carbon (SOC) detection, satellite imagery, machine learning algorithms, multilayer perception model, feature importance, soil property predicting

## Abstract

We utilized remote sensing and ground cover data to predict soil organic carbon (SOC) content across a vast geographic region. Employing a combination of machine learning and deep learning techniques, we developed a novel data fusion approach that integrated Digital Elevation Model (DEM) data, MODIS satellite imagery, WOSIS soil profile data, and CHELSA environmental data. This combined dataset, named GeoBlendMDWC, was specifically designed for SOC prediction. The primary aim of this research is to develop and evaluate a novel optimization algorithm for accurate SOC prediction by leveraging multi-source environmental data. Specifically, this study aims to (1) create an integrated dataset combining remote sensing and ground data for comprehensive SOC analysis, (2) develop a new optimization technique that enhances both machine learning and deep learning model performance, and (3) evaluate the algorithm’s efficiency and accuracy against established optimization methods like Jaya and GridSearchCV. This study focused on India, Australia, and South Africa, countries known for their significant agricultural activities. We introduced a novel optimization technique for both machine learning and deep neural networks, comparing its performance to established methods like the Jaya optimization technique and GridSearchCV. The models evaluated included XGBoost Regression, LightGBM, Gradient Boosting Regression (GBR), Random Forest Regression, Decision Tree Regression, and a Multilayer Perceptron (MLP) model. Our research demonstrated that the proposed optimization algorithm consistently outperformed existing methods in terms of execution time and performance. It achieved results comparable to GridSearchCV, reaching an R^2^ of 90.16, which was a significant improvement over the base XGBoost model’s R^2^ of 79.08. In deep learning optimization, it significantly outperformed the Jaya algorithm, achieving an R^2^ of 61.34 compared to Jaya’s 30.04. Moreover, it was 20–30 times faster than GridSearchCV. Given its speed and accuracy, this algorithm can be applied to real-time data processing in remote sensing satellites. This advanced methodology will greatly benefit the agriculture and farming sectors by providing precise SOC predictions.

## 1. Introduction

Soil organic matter and soil organic carbon (SOC) play a vital role in determining soil physico-chemical properties, such as soil structure, water retention capacity, and cation exchange capacity (CEC) [1]. There is an increasing demand for efficient and economical techniques that can accurately estimate soil organic carbon (SOC) levels in field settings without causing any damage to the soil. This demand has driven the development of portable or online sensing technologies [2,3]. Accurate spatial measurement of SOC content is crucial for environmental and agricultural applications due to its influence on soil erosion resistance [4,5].

Visible and Near-Infrared Spectroscopy (VNIRS) is widely used in soil research for predicting soil organic carbon (SOC) both in laboratory and field conditions [6,7], enabled by advances in robust, portable detectors [8,9,10]. As a key soil quality indicator, SOC can be reliably measured through VNIRS due to its distinctive near-infrared spectral signature and its effect on soil color [4,11,12]. When calibrated with machine learning and deep learning techniques, VNIRS provides a fast and cost-effective method for SOC estimation in field settings [13,14].

Several studies have highlighted the influence of soil surface factors, such as vegetation coverage, debris, soil wetness, and soil roughness, on SOC prediction [15]. Promising outcomes have been attained in several research endeavors on soil organic carbon (SOC) prediction utilizing remote sensing data, with a specific emphasis on optical data sources such as Sentinel-2 [15,16,17,18,19,20,21], Landsat [22,23,24,25,26,27,28], and MODIS satellite data [29,30,31]. These data sources encompass a wide range of spectral bands, spanning from visible to short-wave infrared, offering richer information for analysis. However, when taking into account real-time data usage and useful applications, the prediction accuracy usually stays below optimal levels. Additionally, because convolutional neural networks (CNNs) are complicated networks, these techniques are frequently computationally costly. Crucially, most researchers focus on small regions, which makes the algorithms and outcomes too specific for wide-ranging real-world applications across various soil types.

Through the integration of WoSIS soil data, MODIS satellite imagery, CHELSA environmental data, and DEM data, we provide a distinctive dataset that enables precise forecasting of soil organic carbon levels using sophisticated machine learning and deep learning methodologies on a much larger scale, which makes our algorithms applicable for practical applications. Additionally, we have employed different optimization approaches such as Jaya optimization to enhance the performance of deep neural networks and GridSearchCV to optimize machine learning algorithms on the dataset. In addition, we have introduced our novel optimization technique, which we have named the JR algorithm (which is a modification of the Jaya [32] and Rao [33] optimization algorithms) and conducted a comprehensive comparison analysis of its outcomes and average execution time to run all three optimization techniques for each different model. We conducted a comprehensive comparison of the JR algorithm with other optimization techniques across three regions: India, Australia, and South Africa. These regions were selected due to their significant agricultural activities.

These three regions represent vastly different agricultural landscapes, each with unique farming practices, crop varieties, and technological adoption levels. India predominantly features mixed crop–livestock farming and rice–wheat rotation systems, while Australia is known for large-scale mechanized farming and dryland agriculture, and South Africa combines commercial farming with subsistence practices.

The types of crops planted in these regions are also diverse due to different climatic conditions, local demand, and cultural preferences. For example, common crops by region include rice, wheat, pulses, and sugarcane in India; wheat, barley, and cotton in Australia; and maize, wheat, sugarcane, and sunflowers in South Africa.

The three regions exhibit diverse soil compositions that significantly influence carbon sequestration patterns, with India featuring alluvial and black cotton soils affected by high temperatures, Australia containing red-brown earths and sandy soils limited by low rainfall, and South Africa presenting a mix of sandy loams and clay-rich soils impacted by variable rainfall. The interaction between these soil types, environmental conditions, and farming practices creates complex systems affecting carbon sequestration potential, making it crucial to understand these relationships for developing effective regional carbon management strategies.

Another significant factor in choosing these three nations is their varied farming practices. In India, the average farm size is 1.08 hectares, meaning there is less access to expensive technology and family farming is common, leading to lower labor costs. Additionally, there is a heavy reliance on manual labor and monsoon rains. In Australia, the average farm size is 4000 hectares, featuring advanced GPS-guided machinery but with high labor costs. Corporate farming is common and strict water-efficient allocation systems are present. Between these two extremes lies South Africa, with moderate farm sizes and intermediate levels of technological adoption and labor costs, but where water scarcity remains a major challenge. Integrating data from these three nations provides a detailed view of agricultural practices, from traditional to highly mechanized farming systems, which aligns with our research aim of understanding global agricultural diversity and its impact on productivity.

This study encompasses three distinct continental climates: South Asia’s monsoon-driven climate, Australia’s predominantly arid and semi-arid conditions, and South Africa’s varied subtropical climate zones. This climatic diversity presents both advantages and limitations. While it enables the development of robust models that account for various environmental conditions, it may also limit the models’ direct applicability to regions with significantly different climatic patterns, such as the Mediterranean or tropical rainforest zones. Future research should consider validation studies in other geographic contexts to assess and adapt the models’ predictive capabilities.

The WoSIS data provide detailed soil profiles, including SOC information, while MODIS data capture vegetation and land cover. CHELSA offers environmental variability data, and DEM data provide elevation information.

Our findings demonstrate that the JR algorithm significantly outperforms other optimization strategies in optimizing models using our dataset.

## 2. Materials and Methods

The WOSIS dataset offers information on soil profiles, which is critical for comprehending soil properties and how they could affect vegetation. The Normalized Difference Vegetation Index (NDVI), a measure of vegetation density and productivity, is part of the MODIS NDVI dataset and provides insights into vegetation health. Precipitation and temperature information from the CHELSA dataset is crucial for establishing the general climate and its potential effects on vegetation. The DEM dataset provides information on topography, which can also impact vegetation growth and distribution.

### 2.1. Study Area

**Figure 1:**
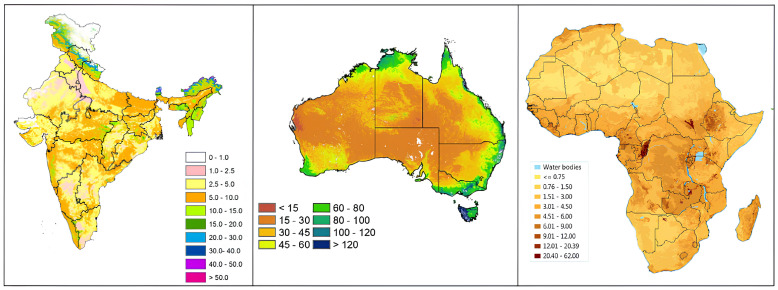
Soil organic carbon content by region in tons-per-hectare (ton/ha) in India [34], Australia [35], and Africa [36] respectively.

The study area encompasses locations in India, Australia, and South Africa, selected for their significant agricultural development. The spatial distribution of SOC content across these regions is illustrated in Figure 1, highlighting distinct patterns across these geographically diverse locations. Soil organic carbon (SOC) is vital for agricultural sustainability in India, Australia, and South Africa, influencing soil fertility, moisture retention, and ecosystem health. In India, climate and land use factors significantly impact SOC levels, affecting agricultural productivity and resilience. Australia faces unique challenges due to limited rainfall and diverse soil compositions, making SOC crucial for maintaining soil health and drought resistance. South Africa’s agriculture-dependent economy relies heavily on healthy soils, which are influenced by climatic variations.

We created a comprehensive dataset by combining environmental data from various sources to gain a holistic understanding of ecosystems. By integrating these data into predictive models, we aim to forecast SOC levels and support informed decision-making for sustainable agriculture and environmental conservation in these regions.

### 2.2. WoSIS Dataset: Soil Profiling Data

The World Soil Information Service (WoSIS) is a resource that offers comprehensive details on soil traits and qualities. It contains information about the characteristics of the soil profile, such as texture, pH, amount of organic matter, and nutrient levels. Automated techniques confirmed WoSIS layer depth consistency (e.g., consecutive increases in upper and lower depth reported for each layer down the profile). International standards report that depth increments are “measured from the surface, including organic layers and mineral covers” [37]. We used the WoSIS—Snapshot 2019 dataset to obtain soil profiles from India, Australia, and Africa, as well as information on their characteristics, such as organic carbon, clay, and silt.

### 2.3. MODIS NDVI: Normalized Difference Vegetation Index

The Normalized Difference Vegetation Index (MODIS NDVI) is a metric for the density and health of vegetation. A Chinese study employed ten-year MODIS MCD12Q2 phenology variables to anticipate SOC using a CNN model in the Anhui region. The CNN took on random forest (RF) in three environmental parameters. Land surface phenology variables and natural environmental parameters enhanced the CNN’s prediction accuracy by 5.57% RMSE and 31.29% $R^{2}$ [38]. By analyzing vegetation cover and land productivity, these data can be combined with soil information to create more accurate predictions of soil carbon levels.

### 2.4. CHELSA: For Precipitation and Temperature

The Climatologies at High Resolution for the Earth’s Land Surface Areas (CHELSA) dataset was created using statistical downscaling of atmospheric temperatures. The precipitation algorithm uses wind fields, valley exposition, and boundary layer height as orographic predictors and corrects bias. The outcome is the 1979–2013 monthly temperature and precipitation climatology [39]. The temperature directly affects the decomposition rates and influences microbial activity too, affecting SOC. At higher temperatures, the microbes rapidly convert the broken down organic carbon to carbon dioxide, creating a negative balance in carbon storage. Precipitation has a relation with SOC too. Optimal rainfall supports plant growth and leaching of organic matter, thus increasing SOC. However, excessive precipitation can lead to waterlogged soil conditions, which reduces decomposition rates due to limited oxygen availability. While slower decomposition typically leads to higher SOC accumulation, in waterlogged conditions, reduced plant growth and poor root development can result in lower organic matter inputs, ultimately leading to decreased SOC levels. CHELSA provides historical temperature and precipitation data trends that help understand these climate–SOC relationships.

### 2.5. DEM: Digital Elevation Model (Topography)

A Digital Elevation Model (DEM) digitally depicts a location’s topography. With additional data sources, forecasts can be strengthened by analyzing how topography influences soil carbon levels. DEM derivatives, multi-temporal Sentinel-1 and Sentinel-2 data, and machine learning methods were utilized to map soil organic carbon and Soil Total Nitrogen content in southern Central Europe. We also evaluated the remote sensing sensors’ predicted soil organic carbon and Soil Total Nitrogen content. According to their analysis, Sentinel-1/2 and DEM derivatives provide the highest forecast accuracy [40].

A DEM’s influence on SOC can be explained through direct topographic effects that significantly shape soil characteristics. The primary topographic factors include slope position and gradient, which fundamentally affect water movement patterns across landscapes. These water movements directly control soil erosion and deposition patterns, where steeper slopes typically experience greater erosion, leading to reduced SOC content, while depressions and valley bottoms accumulate transported organic matter, resulting in higher SOC concentrations. Additionally, elevation plays a crucial role by influencing both temperature and precipitation patterns—key factors that affect vegetation growth and soil formation processes. At higher elevations, lower temperatures typically slow down organic matter decomposition, potentially leading to greater SOC accumulation, while also affecting the type and density of vegetation that can grow. These elevation-dependent climate variations also influence soil moisture regimes, which in turn affect microbial activity and organic matter decomposition rates. Furthermore, topographic position influences soil depth development, with deeper soils generally forming in lower landscape positions, providing a greater capacity for carbon storage. The combination of these topographic influences creates distinct patterns of SOC distribution across landscapes, making a DEM a valuable predictor for understanding and mapping soil carbon stocks.

**Figure 2:**
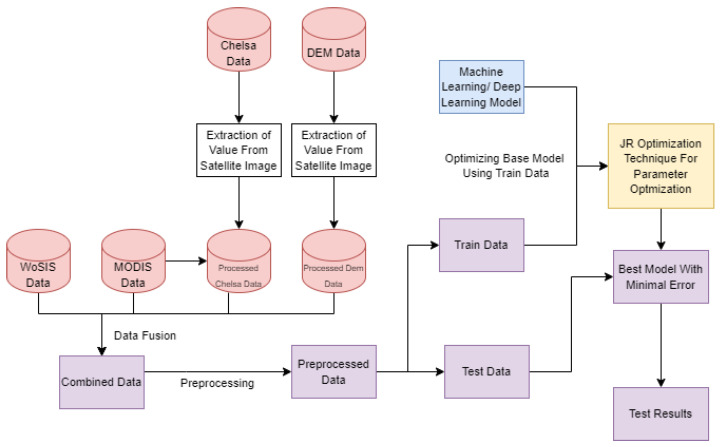
Research workflow.

By merging MODIS satellite pictures, DEM data, WoSIS soil data, and CHELSA environmental data, the accuracy of soil organic carbon (SOC) prediction has been improved. MODIS collects vegetation and land cover data, which are critical for predicting SOC, whereas CHELSA addresses environmental variability. DEM data aid in understanding terrain effects. This study develops a comprehensive SOC distribution dataset incorporating geography, climate, and terrain. The research workflow is shown in the Figure 2.

To assess how various data distributions affected our model’s performance, we created two separate datasets. The first dataset included 681 samples exclusively from India. The second larger, worldwide dataset comprised 71,125 sample points from all three different target regions, soil types, and environmental circumstances. Subsets of each dataset were created, with 80% going toward training, 10% toward testing, and 10% toward validation. By using this method, we were able to investigate how the model’s capacity to generalize soil organic carbon (SOC) predictions was affected by both a single-region dataset and a larger, worldwide dataset. The experimental results revealed that the model trained on the combined global dataset achieved a higher generalization capability across diverse conditions, as reflected in improved accuracy and stability on the validation set.

### 2.6. Novel Approach for Optimizing Models

#### 2.6.1. Algorithm Explanation

We present a new optimization technique that draws inspiration from two well-known optimization algorithms: the Jaya algorithm, a meta-heuristic technique, and the RAO algorithm, known for its simplicity and efficacy, which do not require parameter tuning. The procedure commences by establishing a minimum threshold, a maximum threshold, and a population size. The initial population is randomly created within the specified boundaries. The objective function f(x) is maximized or minimized through iterative updates.

At any iteration *i*, assume there are *m* design variables in each candidate solution and *n* candidate solutions (i.e., population size, k=1,2,…,n). The candidate solution that achieves the best objective function value, $f{\left(x\right)}_{\mathrm{best}}$, is denoted as the best candidate. Similarly, the candidate solution with the worst objective function value, $f{\left(x\right)}_{\mathrm{worst}}$, is denoted as the worst candidate.

If $X_{j,k,i}$ represents the value of the *j*-th variable for the *k*-th candidate solution during the *i*-th iteration, this value is updated according to the following equations:

(1) $$G\_\mathrm{DIFF}=|X_{j,\mathrm{best},i}-X_{j,\mathrm{worst},i}|$$

(2) $$X_{j,k,i}^{\prime }=X_{j,k,i}+r_{1,j,i}\cdot G\_\mathrm{DIFF}+r_{2,j,i}\cdot \left(X_{l,k,i}-X_{j,k,i}\right)$$

Here, $X_{j,\mathrm{best},i}$ is the best value of the *j*-th variable among all *k* candidate solutions during the *i*-th iteration, and $X_{j,\mathrm{worst},i}$ is the worst value of the *j*-th variable among all *k* candidate solutions during the *i*-th iteration. The terms $r_{1,j,i}$ and $r_{2,j,i}$ are random numbers uniformly distributed in the range [0,1]. The variable $X_{j,k,i}^{\prime }$ represents the updated value of the *j*-th variable for the next iteration. The term $X_{l,k,i}$ is the value of a selected variable *l* from the *k*-th solution during the *i*-th iteration, where l≠j and $f\left(X_{l,k,i}\right)>f\left(X_{j,k,i}\right)$ if the problem is one of maximization, or $f\left(X_{l,k,i}\right)<f\left(X_{j,k,i}\right)$ if the problem is one of minimization. If no solution satisfies this condition, $X_{l,k,i}$ is chosen randomly. G_DIFF is the difference between the best solution and worst solution for the current iteration.

The values are updated only if the fitness of the new solution $X_{j,k,i}^{\prime }$ is better than that of the previous solution $X_{j,k,i}$. In simpler terms, we are using the formula proposed to generate a new candidate solution. Then, we are comparing their fitness with the fitness of their parent candidate solutions. If the new candidate solution has a better fitness, we are taking the new candidate solution and replacing it with the parent solution with lower fitness in the next generation. Otherwise, we are keeping the parent solution. This mechanism ensures that the algorithm maintains the best solutions while exploring the search space. This innovative strategy synergizes the advantages of the Jaya and RAO algorithms, utilizing the most favorable and unfavorable candidate solutions to direct the search process while upholding simplicity and speed. The algorithm’s performance is showcased by optimizing machine learning and deep learning models, which are trained on our novel soil organic carbon dataset.

#### 2.6.2. Model Building Methodology Using Optimization Algorithms

We employed the JR optimization algorithm to optimize the hyperparameters of machine learning models and a deep learning model. We conducted a grid search to explore a range of parameter values and evaluate their combined impact on model loss. The JR optimization technique employed a systematic search to identify the parameter configuration that minimized the loss. The algorithm successfully explored the parameter space and found the best configuration, leading to a model that performs better and has less loss.

We conducted a comprehensive study utilizing five distinct machine learning methods: XGBoost [41], LightGBM [42], Gradient Boosting Regressor (GBR) [43], Decision Tree [44], and random forest [45]. These algorithms were applied to our dataset on soil organic carbon. The model parameters were tuned to maximize the $R^{2}$ metric [46] using the JR optimization technique and GridSearchCV. A comparative analysis was conducted to assess the efficacy of the optimization techniques.

In addition, we incorporated a Multilayer Perceptron (MLP) [47,48] model to carry out the prediction task. We optimized the architecture of the MLP by determining the optimal number of hidden units in the dense layers [49,50,51] and selecting the most suitable activation functions [52] using the JR optimization technique to optimize the loss function [53]. In order to make comparisons, we also utilized the Jaya optimization approach to optimize the MLP. We employed the JR optimization algorithm to refine the architecture of a Multilayer Perceptron (MLP) neural network, with the objective of determining the optimal neuron configuration in each layer to achieve a balance of efficiency and accuracy. To comprehensively evaluate the optimization process, we maintained a straightforward MLP architecture: an input layer with 12 neurons, followed by two dense hidden layers. Each hidden layer was succeeded by a dropout layer with a 20% dropout rate to mitigate overfitting, and the model concluded with a single output neuron. The JR optimization revealed that the ideal configuration for our problem included 28 neurons in each hidden layer, resulting in a model with 1205 trainable parameters, effectively balancing model complexity and predictive performance.

The input layer was configured with 12 neurons, exceeding the 8 available input features, as this architecture was found to yield the best performance during optimization. The choice of 12 neurons was exploratory, based on the observation that model accuracy and performance were enhanced with this configuration, likely due to a richer feature representation enabled by the additional neurons.

The parameter count for this model is calculated as follows: The connection between the input layer (12 neurons) and the first hidden layer (28 neurons) contributes 336 weight parameters and 28 biases, totaling 364 parameters. The first hidden layer connects to the second hidden layer (also with 28 neurons), adding 784 weight parameters and 28 biases, totaling 812 parameters. Finally, the second hidden layer connects to the output layer (single neuron), introducing 28 weights and 1 bias, resulting in 29 parameters. Summing all layers, the model has 1205 trainable parameters.

The model was trained using the mean squared error (MSE) loss function to minimize prediction errors, which is appropriate for our target variable, soil organic carbon content, a continuous measure. Weight updates were computed using the Adam optimizer, selected for its efficiency and adaptive learning in regression tasks. Given the continuous nature of the output, a linear activation function was applied to the output neuron, allowing the network to produce predictions across an unrestricted range of real values.

Our research indicates that within the context of deep neural networks, our proposed optimization algorithm not only converges significantly faster than others but also consistently outperforms them in most cases. The machine learning models optimized using our technique achieved performance comparable to those improved with GridSearchCV [54,55]. Notably, our method demonstrated a substantial reduction in computational time, delivering identical results in approximately 20–30 times less time.

**Figure 3:**
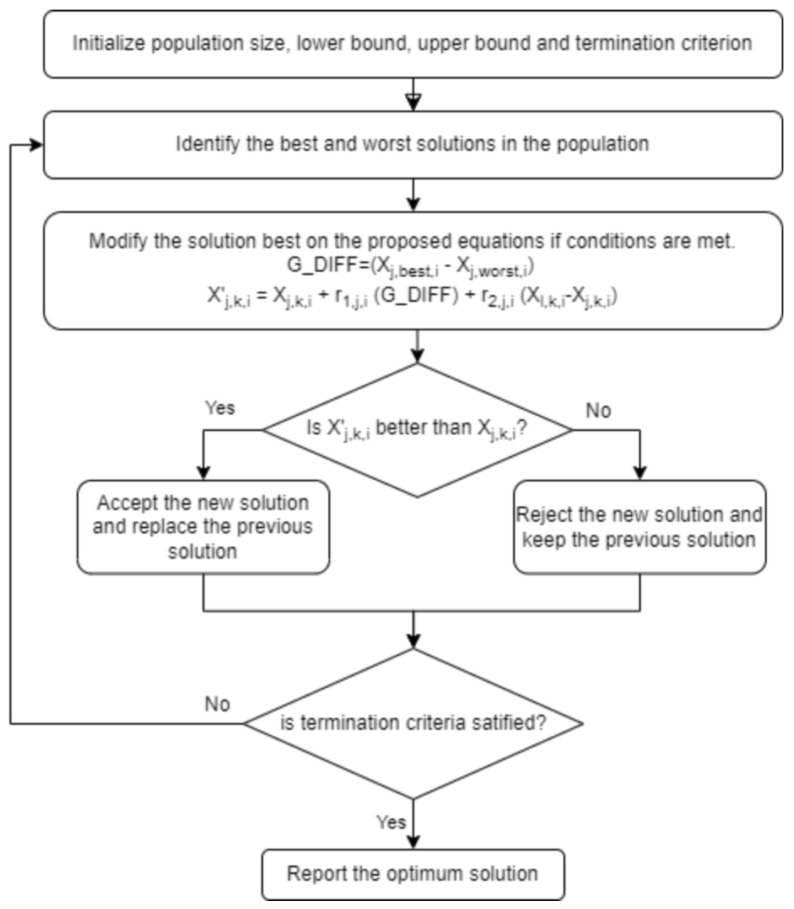
Flowchart of the optimization algorithm.

Therefore, our suggested optimization strategy effectively achieves a compromise between time complexity and performance, providing an efficient and effective method for optimizing machine learning models and deep neural networks. The flowchart in Figure 3 showcases how the optimization algorithm works.

### 2.7. Model Evaluation Parameters

The present study observed field data throughout India, Australia, and Africa to investigate the measure of SOC prediction. The model was built using a mixed signal considering soil and plants. As a result of the experiment, we investigated the impact of WOSIS, MODIS, CHELSA, DEM data when used all together. The model was built and tested with different combinations of input features and among them we selected the best 8 features from the dataset:

- Upper Depth
- Lower Depth
- Average Clay Value
- Average Silt Value
- Annual Temperature
- Annual Precipitation
- DEM
- MOD13A1 006 500m 16 day NDVI

Model performance for various land use types was evaluated using the squared error (MSE), percent root mean square error (%RMSE), and $R^{2}$, which ranges from 0 to 1, representing how closely the observed value matches the regression line that best fits the data or the variance ratio that independent predictors can explain. When $R^{2}$ is very close to 1, the model is very stable and has a high degree of fitting. The model’s predictive power and resilience increase with decreasing MSE and MAE. The accuracy of the model is excellent when %RMSE < 10%, acceptable if 10% < %RMSE < 20%, fair if 20% < %RMSE < 30%, and poor if %RMSE > 30%. %RMSE is dimensionless.

(3) $$R^{2}=1-\frac{\sum_{i=1}^{n}{\left(y_{i}-{\hat{y}}_{i}\right)}^{2}}{\sum_{i=1}^{n}{\left(y_{i}-\bar{y}\right)}^{2}}$$

(4) $$MAE=\frac{1}{n}\sum_{i=1}^{n}\left|y_{i}-{\hat{y}}_{i}\right|$$

(5) $$MSE=\frac{1}{n}\sum_{i=1}^{n}{\left(y_{i}-{\hat{y}}_{i}\right)}^{2}$$

(6) $$\%RMSE=\frac{\sqrt{MSE}}{\bar{y}}\times 100\%$$

*m* is the number of samples, $y_{i}$ is the true value, ${\hat{y}}_{i}$ is the predicted value, and $\bar{y}$ is the average value of *y*.

We compared our proposed JR algorithm with other optimization algorithms based on the accuracy achieved post-optimization under identical optimization parameters, as well as the time required to reach a superior solution. This evaluation highlights the JR algorithm’s effectiveness in achieving high accuracy and efficient convergence relative to other methods.

## 3. Results

### 3.1. Statistical Analysis of SOC Dataset

Table 1 displays the statistical parameters of our processed dataset. The raw dataset was processed by creating box plots for each feature and identifying the data points that deviated significantly from the norm, known as outliers.

Since the raw dataset contained minimal null values, we eliminated them. To address the significant variance in features, we normalized the dataset prior to model training.

**Figure 4:**
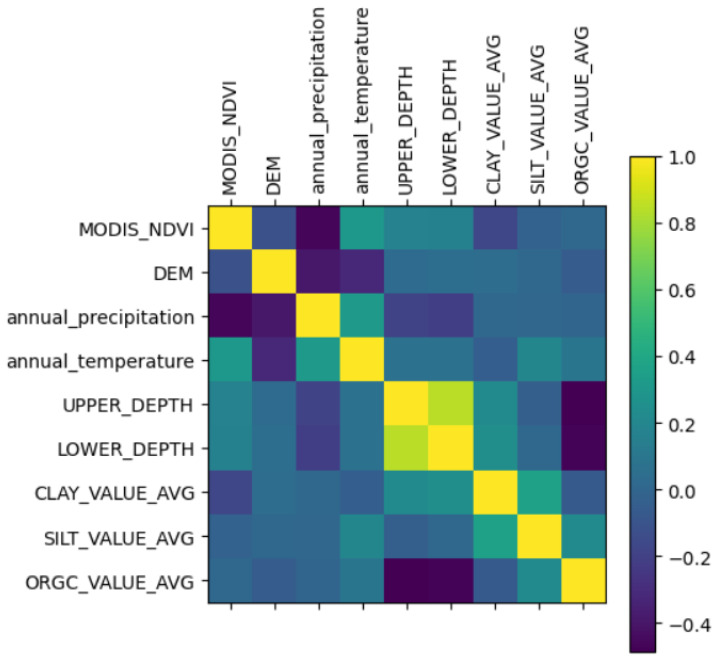
Correlation matrix for the Indian–Australian–African combined dataset. Pearson correlation methodology is used to calculate the correlation values.

Figure 4 depicts the correlation matrix of the dataset. The matrix demonstrates a strong link between the dependent feature, ORGC_VALUE_AVG, and most of the independent features. Pearson correlation methodology was used to calculate the correlation between each of the features of our dataset, which resulted in a value between −1 and 1. The values are unit-less quantities used to describe the amount of correlation between any two features, as can be shown in the correlation matrix. Here, −1 denotes negatively correlated values and 1 denotes positively correlated values. Conversely, the independent traits exhibit minimal correlation as most of the values are between −0.4 and 0.4. This is beneficial for regression situations such as the one currently being discussed. This lack of multicollinearity among the independent variables enhances the reliability and interpretability of the regression models.

### 3.2. Analysis of Model Prediction Results

Table 2 displays the performance of different machine learning models under three conditions: without any optimization technique, with parameter tuning using GridSearchCV, and with the application of JR optimization to reach the highest achievable $R^{2}$ value. All experiments maintained consistent optimization circumstances, which encompassed parameter value ranges and loss functions. The JR algorithm notably improved the $R^{2}$ value for the XGBoost model. The initial XGBoost model, which was not optimized, yielded a $R^{2}$ value of 68.88%. However, the JR algorithm significantly improved its performance, resulting in an astounding $R^{2}$ value of 90.16%. This level of performance is almost on par with the results achieved with GridSearchCV. Furthermore, our system achieved this level of accuracy nearly twenty to thirty times faster than GridSearchCV.

Similarly, the JR algorithm, on Gradient Boosting Regressor (GBR), had a 22.31% higher performance than the GridSearchCV optimization in terms of the $R^{2}$ metric. When evaluated on other models, our approach achieved comparable results to GridSearchCV in significantly less time.

The results for our deep learning Multilayer Perceptron (MLP) model are shown in Table 3. We utilized optimization techniques to optimize the neural network architecture, specifically by adjusting the number of hidden units and the activation functions in the hidden layers, with the goal of maximizing the $R^{2}$ value. Our approach significantly enhanced the performance of the neural network under the same optimization settings.

The coefficient of determination ($R^{2}$) for the MLP model without any optimization was 28.32%, and it improved to 61.34% after optimization using the JR algorithm. By comparison, the Jaya algorithm only achieved a 30.04% $R^{2}$ value on our SOC dataset. As a result, the JR algorithm demonstrated a significant improvement of 33.02% in the $R^{2}$ metric when compared to the unoptimized MLP model. Although the MLP’s overall performance was not exceptional, the JR algorithm greatly improved the results and surpassed the Jaya algorithm in refining the design of the deep neural network. In the Table 4, Table 5, Table 6 and Table 7, we have represented the scatter and residual plot result of each model showcasing the effect of the different optimization techniques.

We graphically show and contrast the scatter plots of the test data for the machine learning models and our deep learning Multilayer Perceptron (MLP) model in Table 4 and Table 6. In the vertical axis, the actual values of the data samples (g/kg) are represented and in the horizontal axis, the corresponding predicted values by the models (g/kg) are represented. Scatter plots aim to visualize data points closely aligned with the (y = x) line, indicating a strong correlation between predicted and actual values. The scatter plots clearly demonstrate that optimization significantly improves results, with data points clustering more closely around the (y = x) line. Notably, the scatter plots generated by the JR algorithm are nearly identical to those produced by GridSearchCV.

Table 5 and Table 7 display the residual plots for both the machine learning models and the deep learning MLP model. In the vertical axis, the values of residuals (g/kg) are represented and in the horizontal axis, the corresponding predicted values (g/kg) are represented. The objective of residual plots is to have residuals (the discrepancies between the observed and predicted values) near the (*y* = 0) line, showing minimal prediction errors and the absence of any systematic bias. The residual plots indicate that the JR optimization algorithm significantly reduces the residuals, bringing them much closer to the (*y* = 0) line. This improvement is more pronounced than that achieved by unoptimized models or those optimized using other techniques.

Scatter plots depict the correlation between observed and forecasted numbers in a visual manner. The proximity of the data points to the (*y* = *x*) line directly correlates with the accuracy of the model’s predictions.

Residual plots display the residuals on the vertical axis and the anticipated values (or occasionally the actual values) on the horizontal axis. Residuals should ideally have a random distribution around the horizontal axis (y=0), indicating that the model has accurately captured all patterns in the data without any systematic errors. The improvements observed in both scatter and residual plots confirm the JR algorithm’s effectiveness in enhancing forecast accuracy and model reliability.

To summarize, the JR algorithm approach has shown significant enhancements in the efficiency of different machine learning and deep learning models when trained on our dataset. It achieves these improvements in a fraction of the time required by other algorithms, making it both computationally economical and successful in real-time data processing situations.

### 3.3. Optimization Time Required by Models

All models were trained using the Google Cloud Platform’s Vertex AI service. Specifically, the instance type employed was e2-standard-8, which provides 8 vCPUs, 32 GB of memory, 128 persistent disks (PDs), a maximum total PD size of 257 tebibytes (TiB), and 16 Gbps of bandwidth.

It was observed that our algorithm was able to reach an optimization faster when compared to GridSearchCV. By experimental means, we were able to calculate the time taken to reach an optimized state in each case, and the machine learning models were optimized at least 20–30 times faster when compared to GridSearchCV. In the case of the Multilayer Perceptron (MLP), the Jaya optimization technique was used as a benchmark to evaluate the effectiveness of our proposed optimization algorithm. While both methods were able to converge to a solution in about 60 iterations, our optimization algorithm achieved superior results by identifying a more optimal neural network structure. This suggests that our method is not only efficient in terms of convergence rate but also more effective in optimizing the architecture for enhanced performance.

**Figure 5:**
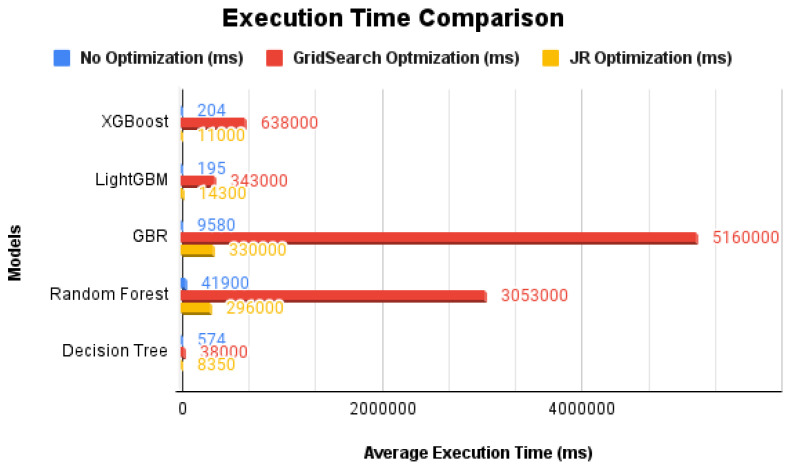
Average execution time comparison in milliseconds between the machine learning models when using different optimization techniques.

In Figure 5, we present a comparative graph illustrating the average execution time required for each algorithm when trained under three different conditions: without any optimization, with GridSearchCV, and with our proprietary optimization algorithm. The execution time chart only considers the machine learning model training.

## 4. Discussion

### 4.1. Performance of Prediction Models

This study assesses prediction models for estimating soil organic carbon (SOC) concentrations using an integrated methodology incorporating data from many sources, such as satellite imagery and field observations. This study specifically focuses on regions in India, Australia, and South Africa. Precise estimations of soil organic carbon (SOC) concentration are essential for effective land management, agricultural practices, and climate change studies to preserve soil health and ensure the proper functioning of ecosystems.

To encompass the wide range of climatic conditions in the research locations, we integrated the Climatologies at High Resolution for the Earth’s Land Surface locations (CHELSA) dataset, which offers detailed information on temperature and precipitation at a high level of accuracy. Understanding SOC dispersion relies heavily on these climate conditions. In addition, Digital Elevation Model (DEM) data were utilized to consider topographic characteristics, which have a substantial impact on soil formation and hydrological processes.

Data preprocessing, including cleaning and filtering, was crucial for ensuring the accuracy of our forecasts. These steps maintained the consistency and quality of the merged dataset by reducing outliers and addressing missing values. This thorough preparation enhanced the reliability of our predictive models.

We assessed the predicted precision of our models and found that our combined methodology surpasses earlier research that only relied on WoSIS, CHELSA, MODIS, and DEM datasets. The utilization of high-resolution satellite data has improved our forecasts, providing a more profound understanding of SOC dynamics and their correlations with environmental parameters and land characteristics.

Compared to traditional grid search methods, the JR algorithm demonstrated significantly faster performance, although with a slight trade-off in accuracy. This speed–accuracy trade-off is crucial in practical applications where rapid results are essential. Our method’s efficiency is particularly valuable for large-scale studies and real-time applications, even if it might not always achieve the highest possible accuracy.

The implications of this study are significant. Accurate estimation of soil organic carbon (SOC) content on a large scale has profound implications for regional policy-making, agricultural decision-making, and soil management practices. Our research provides evidence to support initiatives focused on carbon sequestration, sustainable land management, and soil health conservation. The integration of multiple data sources, especially satellite imagery, is essential for understanding the distribution of SOC and its correlation with environmental variables. This integration offers a reliable tool for future ecological and agricultural assessments

### 4.2. Aerospace Application for Soil Organic Carbon Predictions

Soil organic carbon (SOC) prediction is crucial for aeronautical applications in precision agriculture, sustainable land management, and climate change monitoring. Accurate SOC estimation contributes to climate change mitigation by enhancing our understanding of carbon sequestration. Satellite remote sensing enables comprehensive SOC monitoring, providing high-resolution data that are essential for assessing soil productivity and health. This information helps to reduce environmental impacts and promote sustainable agricultural practices.

To increase prediction accuracy and spatial precision, researchers have created models that use hyperspectral imagery from Sentinel-1 and Sentinel-2 to forecast SOC [56,57,58,59]. Nevertheless, these models frequently prove inadequate for large-scale and real-time applications.

The JR optimization algorithm addresses these limitations by delivering reliable results rapidly. Its speed makes it suitable for real-time SOC monitoring via satellites, allowing for dynamic adjustments based on immediate feedback. This capability is crucial for improving agricultural and environmental management, as it enables quick processing and analysis of satellite data to inform sustainable practices. The ability to process data in near real-time ensures that agricultural decisions can be made promptly, enhancing productivity and reducing ecological impacts.

## 5. Conclusions

In this study, we have developed a novel optimization approach tailored to enhance the prediction of soil organic carbon (SOC) content using data from diverse geographical regions, including India, Australia, and South Africa. Our approach was benchmarked against the traditional grid search method, and the results demonstrated that our method offers significant improvements in computational efficiency while maintaining an acceptable level of prediction accuracy. The integration of multiple data sources, including MODIS NDVI for vegetation indices, CHELSA for climatic variables, DEM for topographical features, and WOSIS for soil profiles, played a critical role in enhancing the model’s prediction capabilities. This comprehensive data integration allowed us to capture the complex interactions between various environmental factors influencing SOC content.

The JR algorithm presents a viable alternative to traditional methods, offering a balanced trade-off between computational efficiency and prediction accuracy. This makes it particularly suitable for large-scale environmental and agricultural applications where timely and reliable SOC predictions are essential.

As demonstrated with various standard machine learning and deep learning methods, the JR optimization algorithm was 10 to 50 times faster than GridSearch. Besides its computational speed, our proposed algorithm produced comparable, and in some cases better, results than GridSearch across performance metrics such as R-squared percentage, Mean Absolute Error percentage, Mean Squared Error percentage, and Relative Mean Squared Error percentage. Additionally, the performance visualizations, including scatter plots and residual plots, were as good as or better than those from previously proposed algorithms. Future research could focus on further refining the algorithm to reduce accuracy trade-offs and exploring its use in other environmental modeling applications.

## Figures and Tables

**Table 1:** Statistical analysis for the Indian–Australian–African combined dataset.

Parameter	Mean	Median	Standard Deviation	Minimum	Maximum	Variance
MOD13A1 006 500m 16 days NDVI	0.48	0.45	0.20	−0.17	0.93	0.04
DEM	237.14	228.59	167.61	−2.76	709.87	28,093.43
Annual Precipitation (mm year^−1^)	188.99	182.00	36.62	95.00	278.00	1341.06
Annual Temperature (°C × 10)	766.66	629.00	458.36	135.00	3975.00	210,102.18
Upper Depth (cm)	18.32	10.00	20.86	0.00	75.00	435.11
Lower Depth (cm)	42.19	30.00	33.87	1.00	156.00	1147.24
Average Clay Value (g/100 gm)	27.30	23.00	19.85	0.00	100.00	394.20
Average Silt Value (g/100 gm)	12.31	11.00	8.32	0.00	38.00	69.22
Average SOC Value (g/kg)	8.13	6.60	5.96	0.00	23.50	35.51

MODIS: Moderate Resolution Imaging Spectroradiometer, NDVI: Normalized Difference Vegetation Index, DEM: Digital Elevation Model, SOC: soil organic carbon.

**Table 2:** Performance metrics of machine learning models for the Indian–Australian–African combined dataset.

Model	Optimization Algorithms	R^2^ (%)	MAE (%)	MSE (%)	RMSE (%)
Xgboost	No Algorithm	68.88	30.08	16.83	41.03
	GridSearch	94.71	9.83	2.86	16.91
	JR Algorithm	90.16	14.88	5.31	23.06
Random Forest	No Algorithm	89.90	14.75	5.53	23.52
	GridSearch	92.36	12.89	4.13	20.33
	JR Algorithm	90.01	14.65	5.41	23.26
LightGBM	No Algorithm	60.11	34.59	21.57	46.45
	GridSearch	90.50	15.07	5.14	22.67
	JR Algorithm	88.01	17.08	6.48	25.46
Decision Tree	No Algorithm	80.77	14.16	10.37	32.20
	GridSearch	89.75	6.08	5.54	23.54
	JR Algorithm	83.86	9.18	8.73	29.55
GBR	No Algorithm	50.11	39.25	26.98	51.95
	GridSearch	67.01	31.23	17.85	42.25
	JR Algorithm	89.32	15.63	5.77	24.03

R^2^ (%): R-squared percentage, MAE (%): Mean Absolute Error percentage, MSE (%): Mean Square Error percentage, RMSE (%): Root Mean Square Error percentage.

**Table 3:** Performance metrics of deep learning models for the Indian–Australian–African combined dataset.

Model	Optimization Algorithms	R^2^ (%)	MAE (%)	MSE (%)	RMSE (%)
MLP	No Algorithm	28.32	61.52	139.92	118.29
	Jaya Optimization	30.04	55.63	71.56	84.59
	JR Algorithm	61.34	51.59	67.03	81.87

R^2^ (%): R-squared percentage, MAE (%): Mean Absolute Error percentage, MSE (%): Mean Square Error percentage, RMSE (%): Root Mean Square Error percentage.

**Table 4:** Model performance visualizations of scatter plots of machine learning models.

Model	Unoptimized Scatter Plot	GridSearch Scatter Plot	JR Algorithm Scatter Plot
Xgboost	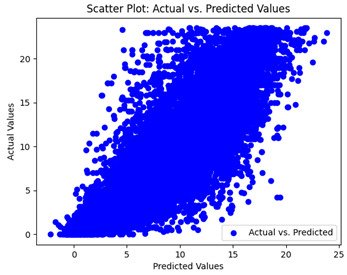	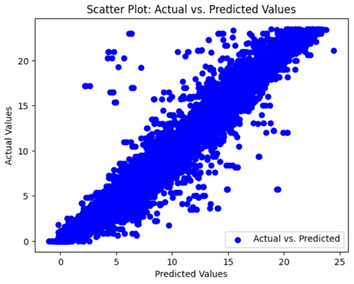	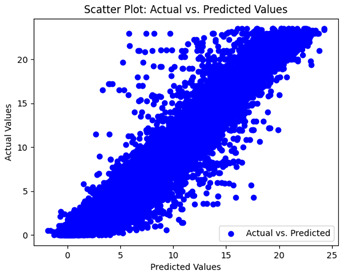
LightGBM	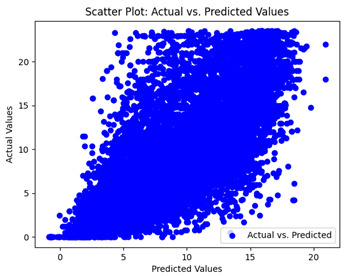	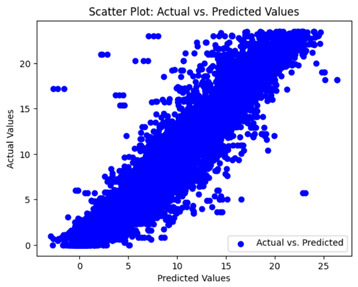	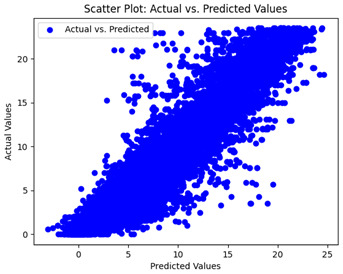
GBR	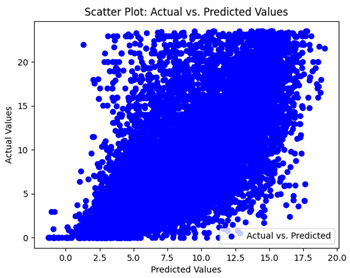	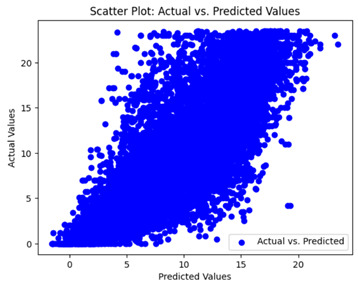	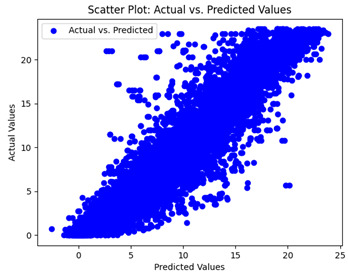
Random Forest	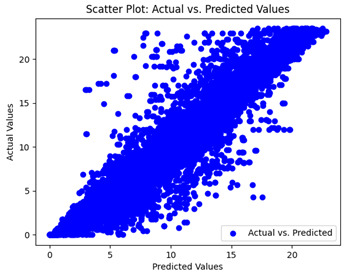	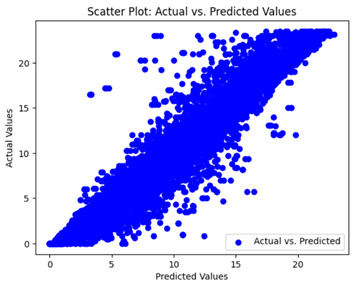	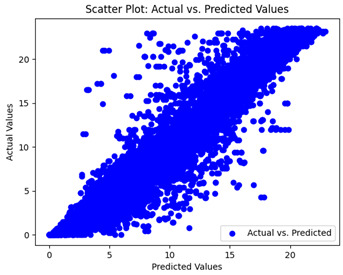
Decision Tree	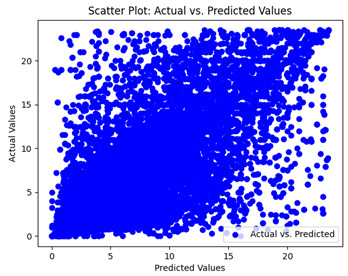	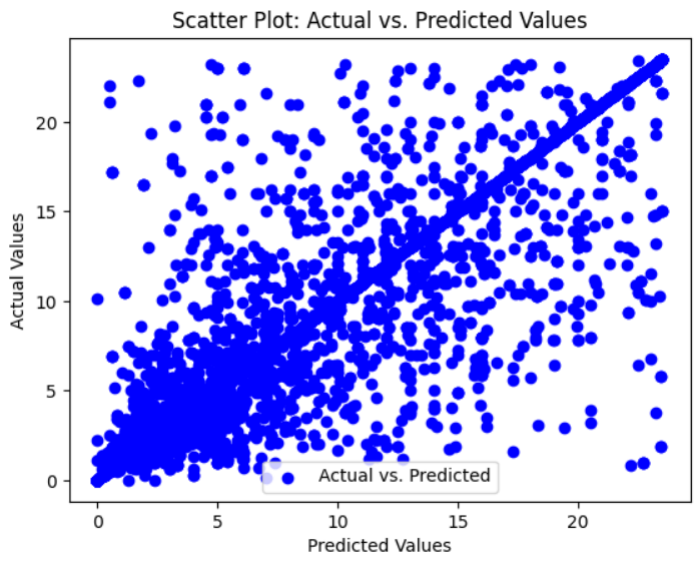	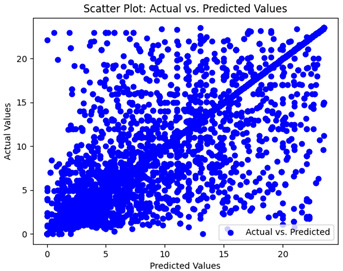

**Table 5:** Model performance visualizations of residual plots of machine learning models.

Model	Unoptimized Residual Plot	GridSearch Residual Plot	JR Algorithm Residual Plot
Xgboost	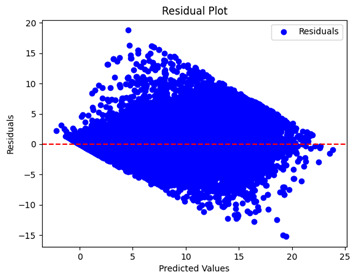	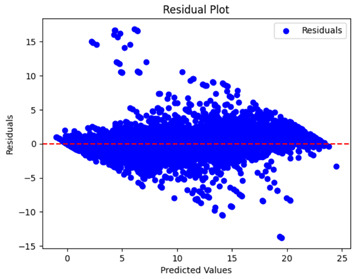	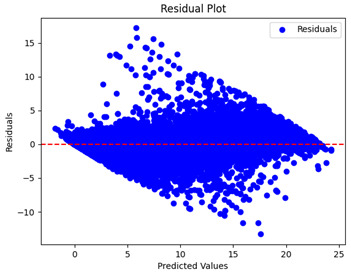
LightGBM	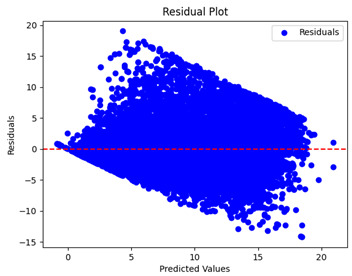	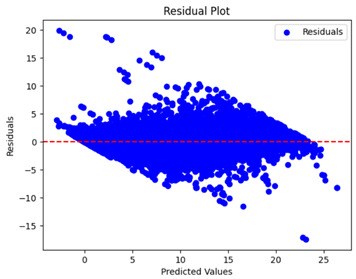	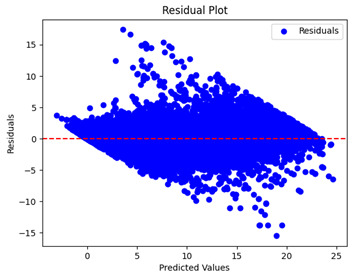
GBR	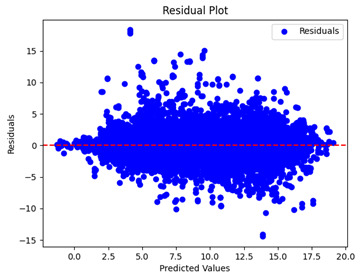	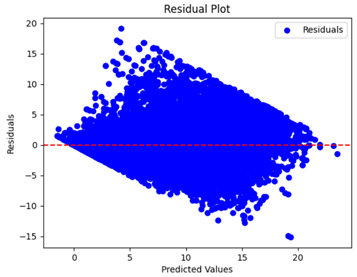	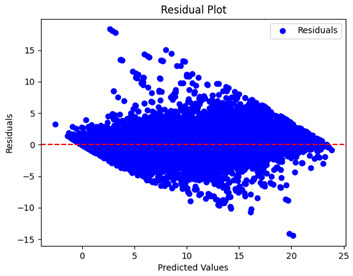
Random Forest	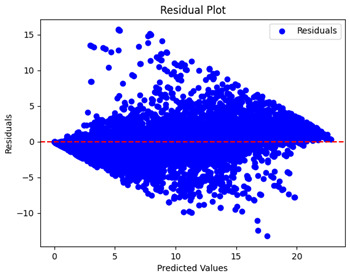	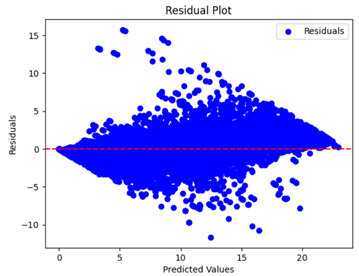	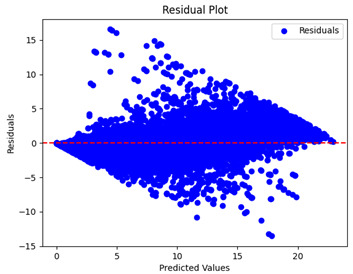
Decision Tree	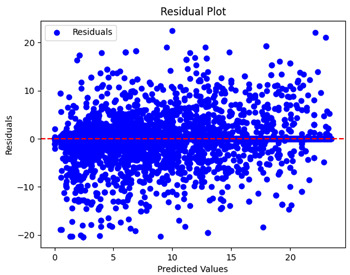	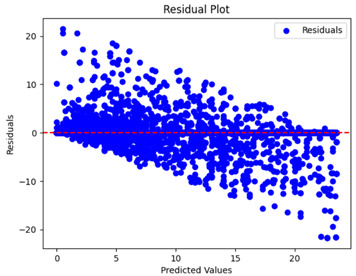	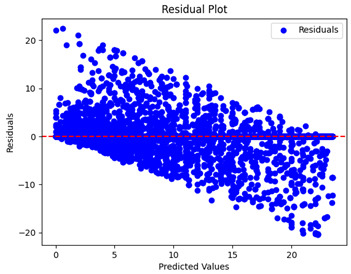

**Table 6:** Model performance visualizations of scatter plots of deep learning models.

Model	Unoptimized Scatter Plot	Jaya Scatter Plot	JR Algorithm Scatter Plot
MLP	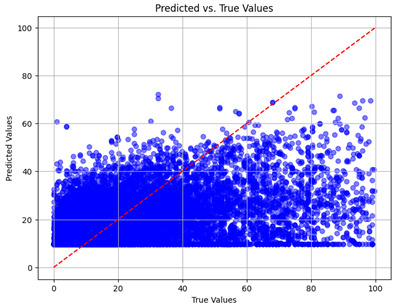	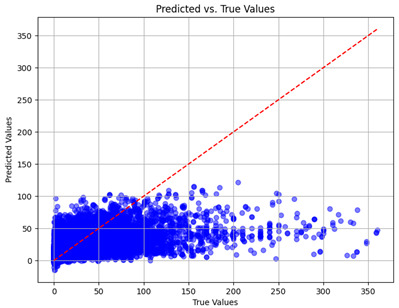	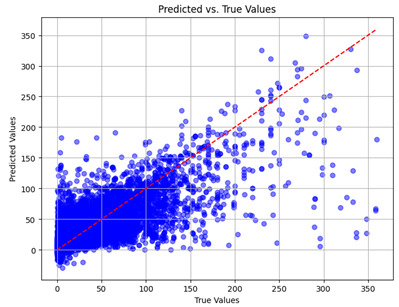

**Table 7:** Model performance visualizations of residual plots of deep learning models.

Model	Unoptimized Residual Plot	Jaya Residual Plot	JR Algorithm Residual Plot
MLP	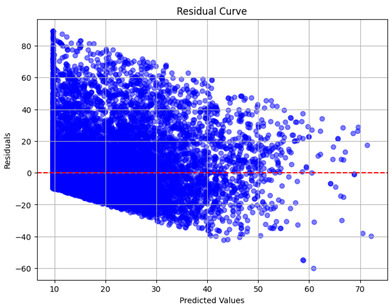	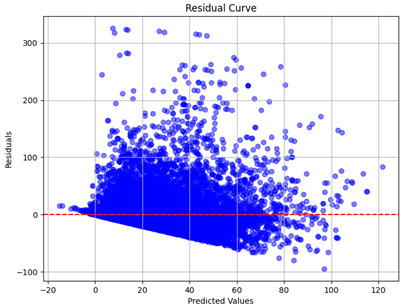	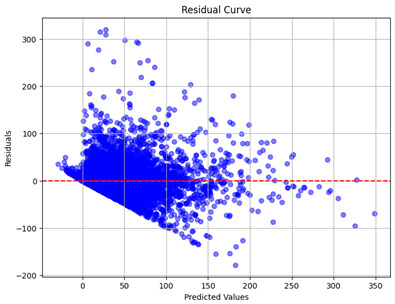

## Data Availability

We used DEM, CHELSA, WoSIS, and MODIS data from their respective websites. WoSIS data were obtained from https://www.isric.org/explore/wosis (accessed on 16 January 2023), MODIS data were obtained from https://modis.gsfc.nasa.gov/data/ (accessed on 16 January 2023), CHELSA data were obtained from https://chelsa-climate.org/ (accessed on 16 January 2023), and DEM (Digital Elevation Model) (topography) data were obtained from http://hydro.iis.u-tokyo.ac.jp/~yamadai/MERIT_DEM/ (accessed on 16 January 2023).
